# Exotic superfluidity and pairing phenomena in atomic Fermi gases in mixed dimensions

**DOI:** 10.1038/s41598-017-13321-3

**Published:** 2017-10-11

**Authors:** Leifeng Zhang, Yanming Che, Jibiao Wang, Qijin Chen

**Affiliations:** 1 Department of Physics and Zhejiang Institute of Modern Physics, Zhejiang University, Hangzhou, Zhejiang, 310027 China; 2 Synergetic Innovation Center of Quantum Information and Quantum Physics, Hefei, Anhui 230026 China; 3 TianQin Research Center & School of Physics and Astronomy, Sun Yat-Sen University (Zhuhai Campus), Zhuhai, Guangdong, 519082 China; 4 James Franck Institute, University of Chicago, Chicago, Illinois 60637 USA

## Abstract

Atomic Fermi gases have been an ideal platform for simulating conventional and engineering exotic physical systems owing to their multiple tunable control parameters. Here we investigate the effects of mixed dimensionality on the superfluid and pairing phenomena of a two-component ultracold atomic Fermi gas with a short-range pairing interaction, while one component is confined on a one-dimensional (1D) optical lattice whereas the other is in a homogeneous 3D continuum. We study the phase diagram and the pseudogap phenomena throughout the entire BCS-BEC crossover, using a pairing fluctuation theory. We find that the effective dimensionality of the non-interacting lattice component can evolve from quasi-3D to quasi-1D, leading to strong Fermi surface mismatch. Upon pairing, the system becomes effectively quasi-two dimensional in the BEC regime. The behavior of *T*_*c*_ bears similarity to that of a regular 3D population imbalanced Fermi gas, but with a more drastic departure from the regular 3D balanced case, featuring both intermediate temperature superfluidity and possible pair density wave ground state. Unlike a simple 1D optical lattice case, *T*_*c*_ in the mixed dimensions has a constant BEC asymptote.

## Introduction

Ultracold atomic gases have been under active investigation in the past decades with their remarkable tunability in terms of interaction, population and mass imbalance^1,2^, and so on. They have provided an ideal platform for simulating existing and engineering exotic physical systems. Therefore, besides the atomic and molecular physics community, they have attracted a lot of attentions from other fields of physics, including condensed matter, nuclear matter, color superconductivity, etc. In particular, they can be put in an optical lattice^3^, with variable lattice depth and spacing, which controls the hopping integral between neighboring lattice sites. This provides an exciting opportunity for studying exotic many-body phenomena caused by tuning the dimensionality^4,5^. Among others, of great interest are fermion pairing and related superfluid phenomena in mixed dimensions^6,7^.

Recently, Lamporesi *et al*.^8^ has successfully obtained a mixed-dimensional system with a Bose-Bose mixture of ^41^K–^87^Rb using a species-selective one-dimensional (1D) optical lattice technique; only ^41^K atoms feel the lattice potential, leaving ^87^Rb atoms moving freely in the 3D continuum. They observed a series of resonances in the mixed dimensions. Motivated by this experiment, there have been theoretical investigations of the BCS–Bose-Einstein condensation (BEC) crossover in Fermi gases in mixed dimensions. Iskin and coworkers^9^ investigated the phase diagrams of equal population fermion mixtures at zero temperature *T* using a strict mean-field approach and found the phase diagram in some ways similar to the Sarma state in a usual 3D Fermi gas with a population imbalance. In order to address real experiments, studies of phase diagrams at finite temperatures are necessary. However, so far only preliminary study of very limited cases at the finite temperature has be reported in the literature^10^.

In this paper, we explore systematically the effects of mixed dimensionality on the pairing and superfluidity at finite temperatures in two-component ultracold atomic Fermi gases. Due to the high complexity caused by multiple tunable parameters, here we restrict ourselves to the population balanced case with equal masses, in order to single out the effects of the dimensionality mismatch. For the same reason, here we will not consider possible Fulde-Ferrell-Larkin-Ovchinnikov (FFLO) states^11,12^ and phase separation, leaving them to future studies.

We shall consider the same dimensionality setting as in the experiment of ref.^8^, and refer to the lattice and 3D continuum components as spin up and spin down, respectively.

To address the finite temperature effects, we use an existing pairing fluctuation theory, which includes self-consistently the contributions of finite momentum pairs^1,13^, and has been applied to address multiple experiments^14^. We study the behavior of the superfluid transition temperatures *T*_*c*_ as a function of interaction strength throughout the entire BCS-BEC crossover with a varying optical lattice spacing *d* and tunneling matrix element *t*. We find that this non-polarized mixed-dimensional finite *T* result share features in common with a polarized Fermi gas in a simple 3D continuum^15,16^. Our results show that the closest match between the Fermi surfaces of the two pairing components occurs near $t/{E}_{F}=1$ and *k*_*F*_ *d* = 1. (Here the Fermi momentum *k*_*F*_ and energy ${E}_{F}\equiv {\hslash }^{2}{k}_{F}^{2}/\mathrm{(2}m)$ are defined via the 3D component). Deviation from these parameters lead to drastic Fermi surface mismatch, and the resulting phase diagrams can become quite different from their counterpart of the polarized Fermi gases in regular 3D continuum. For a large range of parameters of *d* and *t*, the superfluid phase in the unitary regime may extend all the way down to *T* = 0, allowing a zero *T* superfluid ground state. This is distinct from the population imbalanced Fermi gas case in regular 3D continuum, where an arbitrarily small but finite population imbalance is sufficient to destroy superfluidity at zero *T* at unitarity.

## Theoretical Formalism

We use the same formalism as given in ref.^15^, which is now adapted for the mixed dimensions. For the lattice dimension, we use a one-band nearest-neighbor tight-bind lattice model, and thus the dispersions for the two components are ${\xi }_{{\bf{k}}\uparrow }={{\bf{k}}}_{\perp }^{2}/2m+2t\mathrm{[1}-\,\cos ({k}_{z}d)]-{\mu }_{\uparrow }$ and ${\xi }_{{\bf{k}}\downarrow }={{\bf{k}}}^{2}/2m-{\mu }_{\downarrow }$. Here ${{\bf{k}}}_{\perp }\equiv ({k}_{x},{k}_{y})$, where ${\mu }_{\sigma }$ (with $\sigma =\uparrow ,\downarrow$) are the fermionic chemical potentials. The one-band assumption is appropriate when the lattice band gap is large compared to Fermi energy *E*_*F*_, which may be realized experimentally via a large confining trapping frequency ${\omega }_{z}\gg {E}_{F}$. Indeed, similar one-band models have been used throughout condensed matter theory studies, e.g., in various high *T*_*c*_ superconductivity theories and negative-*U* Hubbard models^17^. (In particular, the typical condensation energy per particle is much less than *E*_*F*_, even at unitarity, and thus not enough to compensate the energy cost for a lattice fermion to occupy the excited bands). As usual, we consider an *s*-wave short range pairing interaction. The bare fermion Green’s functions are given by ${G}_{0\sigma }^{-1}(K)=i{\omega }_{n}-{\xi }_{{\bf{k}}\sigma }$. We refer the readers to ref.^15^ for convention on the four-vector notations and Matsubara frequencies.

Following ref.^15^, the noncondensed pair contributions to the self-energy in the superfluid phase can be well approximated as ${\Sigma }_{pg,\sigma }(K)\approx [{\sum }_{Q}{t}_{pg}(Q)]{G}_{0\bar{\sigma }}(-K)$, in the same form as the superconducting self energy ${{\rm{\Delta }}}_{sc}(K)$, after defining a pseudogap parameter ${{\rm{\Delta }}}_{pg}$ via ${{\rm{\Delta }}}_{pg}^{2}\equiv -{\sum }_{Q}{t}_{pg}(Q)$, where *t*(*Q*) is the pairing *T* matrix, and $\bar{\sigma }=-\sigma$. Then we obtain a total self-energy in the BCS form ${\sum }_{\sigma }(K)=-{{\rm{\Delta }}}^{2}{G}_{0\bar{\sigma }}(-K)$, where ${{\rm{\Delta }}}^{2}={{\rm{\Delta }}}_{sc}^{2}+{{\rm{\Delta }}}_{pg}^{2}$. This immediately leads to the full Green’s functions

1 $$\begin{array}{ccc}{G}_{\sigma }(K) & = & \frac{{u}_{{\bf{k}}}^{2}}{i{\omega }_{n}-{E}_{{\bf{k}}\sigma }}+\frac{{v}_{{\bf{k}}}^{2}}{i{\omega }_{n}+{E}_{{\bf{k}}\bar{\sigma }}},\quad |{k}_{z}| < \frac{\pi }{d}\\ {G}_{\downarrow }(K) & = & \frac{1}{i{\omega }_{n}-{\xi }_{{\bf{k}}\downarrow }},\quad |{k}_{z}| > \frac{\pi }{d}\end{array}$$

where ${u}_{{\bf{k}}}^{2}=\mathrm{(1}+{\xi }_{{\bf{k}}}/{E}_{{\bf{k}}})/\mathrm{2,}{v}_{{\bf{k}}}^{2}=\mathrm{(1}-{\xi }_{{\bf{k}}}/{E}_{{\bf{k}}})/2$, ${E}_{{\bf{k}}}=\sqrt{{\xi }_{{\bf{k}}}^{2}+{{\rm{\Delta }}}^{2}}$, and ${E}_{{\bf{k}}\sigma }={E}_{{\bf{k}}}+{\zeta }_{{\bf{k}}\sigma }$, ${\xi }_{{\bf{k}}}=({\xi }_{{\bf{k}}\uparrow }+{\xi }_{{\bf{k}}\downarrow })/2$, ${\zeta }_{{\bf{k}}\sigma }=({\xi }_{{\bf{k}}\sigma }-{\xi }_{{\bf{k}}\bar{\sigma }})/2$. Note that ${k}_{z\uparrow }$ has been restricted to within the first Brillouin zone, $[-\pi /d,\pi /d]$, due to the lattice periodicity.

With ${n}_{\sigma }={{\rm{\Sigma }}}_{K}{G}_{\sigma }(K)$, we obtain the total atomic number density $n={n}_{\uparrow }+{n}_{\downarrow }$ and the difference $\delta n={n}_{\uparrow }-$ ${n}_{\downarrow }=0$ as

2 $$n=2\sum _{{\bf{k}}}\,[{v}_{{\bf{k}}}^{2}+\bar{f}({E}_{{\bf{k}}})\frac{{\xi }_{{\bf{k}}}}{{E}_{{\bf{k}}}}]+\sum _{|{k}_{z}| > \pi /d}f({\xi }_{{\bf{k}}\downarrow }),$$

3 $$0=\sum _{{\bf{k}}}[f({E}_{{\bf{k}}\uparrow })-f({E}_{{\bf{k}}\downarrow })]-\sum _{|{k}_{z}| > \pi /d}f({\xi }_{{\bf{k}}\downarrow }),$$

where $f(x)$ is the Fermi distribution function, and the average $\bar{f}(x)\equiv {\sum }_{\sigma }f(x+{\zeta }_{{\bf{k}}\sigma })/2$. In contrast to the counterparts in the pure 3D continuum case, there is an extra term of the 3D component in these equations, which has been overlooked in refs^9,10^. When the Fermi energy *E*_*F*_ is lower than the lattice bandwidth 4*t*, its contribution is small. However, its contribution will become large when *t* is small, which is relevant to most 1D optical lattices in experiment as of today.

After Nishida and Tan^6^, we use an effective *s*-wave scattering length *a* in the presence of the mixed dimensionality to characterize the interaction strength between fermions, via the Lippmann-Schwinger relation ${g}^{-1}=m/4\pi a-{\sum }_{{\bf{k}}}1/2{\epsilon }_{{\bf{k}}}$. Here ${\epsilon }_{{\bf{k}}}=({\epsilon }_{{\bf{k}}\uparrow }+{\epsilon }_{{\bf{k}}\downarrow })/2$, with ${\epsilon }_{{\bf{k}}\sigma }={\xi }_{{\bf{k}}\sigma }+{\mu }_{\sigma }$. Note that this scattering length in necessarily different from that defined in ordinary 3D or 2D continuum, and is relevant to the actual scattering length in the presence of the optical lattice, via, e.g., the binding energy ${\varepsilon }_{B}={\hslash }^{2}/2{m}_{r}{a}^{2}$ in the BEC regime. In this way, the divergance of the scattering length *a* corresponds to the threshold interaction strength *g*_*c*_ for two fermions to form a zero binding energy bound state in the mixed dimensions, and where the actual *s*-wave scattering phase shift is *π*/2, i.e., the unitary scattering. In the superfluid state, the Thouless criterion leads to the gap equation

4 $$\frac{m}{4\pi a}=\sum _{{\bf{k}}}\,[\frac{1}{2{\epsilon }_{{\bf{k}}}}-\frac{1-2\bar{f}({E}_{{\bf{k}}})}{2{E}_{{\bf{k}}}}].$$

To better reflect the lattice contribution, we may deduce an effective mass, *m*_*eff*_, from the trace of the inverse mass tensor, $\frac{1}{{m}_{eff}}=\frac{5}{6m}+\frac{1}{3}t{d}^{2}$, and then define an effective scattering length *a*_*eff*_ such that $\frac{m}{4\pi a}=\frac{{m}_{eff}}{4\pi {a}_{eff}}$, or $\frac{1}{{k}_{F}{a}_{eff}}=\frac{1}{{k}_{F}a}\frac{m}{{m}_{eff}}$. In comparison with scattering length *a*, *a*_*eff*_ reflects better the actual scattering length that can be measured experimentally^8^.

The inverse *T* matrix can be expanded as ${t}_{pg}^{-1}(Q)\approx {Z}_{1}{(i{{\rm{\Omega }}}_{{\rm{l}}})}^{2}+Z(i{{\rm{\Omega }}}_{l}-{{\rm{\Omega }}}_{{\bf{q}}})$ in the superfluid phase^1^, where ${{\rm{\Omega }}}_{{\bf{q}}}={q}_{\parallel }^{2}/2{M}_{\parallel }^{\ast }+{q}_{z}^{2}/2{M}_{z}^{\ast }$, with ${M}_{\parallel }^{\ast }$ and ${M}_{z}^{\ast }$ denoting the anisotropic effective pair masses in the long wavelength limit. Here we align the optical lattice in the $\hat{z}$ direction, so that ${{\bf{q}}}_{\parallel }$ and ${q}_{z}$ are the in-plane and out-of-plane pair momenta, respectively. The coefficients *Z*, *Z*_1_, $\mathrm{1/}{M}_{\parallel }$ and $\mathrm{1/}{M}_{z}$ can be computed from straightforward Taylor expansion of the pair susceptibility at $({\rm{\Omega }},{\bf{q}})=0$. It follows that the pseudogap contribution

5 $${{\rm{\Delta }}}_{pg}^{2}=\sum _{{\bf{q}}}\frac{b({\mathop{{\rm{\Omega }}}\limits^{ \sim }}_{q})}{Z\sqrt{1+4\frac{{Z}_{1}}{Z}{{\rm{\Omega }}}_{{\bf{q}}}}}\,,$$

where $b(x)$ is the Bose distribution function and ${\tilde{{\rm{\Omega }}}}_{{\rm{q}}}=Z\{\sqrt{1+4{Z}_{1}{{\rm{\Omega }}}_{{\rm{q}}}/Z}-1\}/2{Z}_{1}$ is the pair dispersion. When ${Z}_{1}\ll Z$, we have ${\tilde{{\rm{\Omega }}}}_{{\rm{q}}}\approx {{\rm{\Omega }}}_{{\rm{q}}}$. The integral over *q*_*z*_ should be restricted to the first Brillouin Zone, $|{q}_{z}| < \pi /d$, since in principle, Ω_q_ will acquire periodicity in *q*_*z*_ as determined by the optical lattice. To a good approximation, one may write ${{\rm{\Omega }}}_{{\bf{q}}}={q}_{\parallel }^{2}/2{M}_{\parallel }^{\ast }+2{t}_{B}\mathrm{[1}-\,\cos ({q}_{z}d)]$, with ${t}_{B}=1/\mathrm{(2}{M}_{z}^{\ast }{d}^{2})$. We have checked numerically that using this band dispersion would only cause slight quantitative difference in *T*_*c*_, as one can see from Supplementary Fig. S2.

Equations (2)–(5) form a closed set, which will be used to solve for the superfluid transition temperature *T*_*c*_ (and the pseudogap Δ_*pg*_ and chemical potentials at *T*_*c*_), by setting the order parameter ${{\rm{\Delta }}}_{sc}=0$. In the superfluid phase, they can be used to solve for various gap parameters as well as corresponding chemical potentials as a function of *T*.

The deep BEC regime can be worked out analytically, where everything is small compared with |*μ*|. Equation (3) drops out, and we obtain

6a $$\mu =-[\frac{1}{4m}{(\frac{\pi }{d})}^{2}+2t]{{\rm{e}}}^{\frac{d}{a}-2C}\,,$$

6b $$n=-\frac{m{\Delta }^{2}}{4\pi \mu d},\quad \quad \Delta =\sqrt{\frac{-4\pi \mu dn}{m}},$$

6c $$\frac{1}{2{M}_{\parallel }^{\ast }}=\frac{1}{2{M}_{z}^{\ast }}=\frac{1}{4m}\,,$$

where the constant $C=\frac{d}{\pi }{\int }_{0}^{\pi /d}\frac{{k}_{z}^{2}+2mtd{k}_{z}\,\sin ({k}_{z}d)}{{k}_{z}^{2}+4mt[1-\,\cos ({k}_{z}d)]}{\rm{d}}{k}_{z}$ only depends on $t,d,m$, and takes the value between 0.7 and 1.0. It is interesting to note that, from Eq. (6c), the effective pair mass M* approaches 2*m* in both in-plane and out-of-plane directions. As a consequence, *T*_*c*_ for all cases will approach roughly the same BEC asymptote, which depends weakly on the lattice constant *d*. This should be contrasted to the counterpart case in which the *z* direction is a lattice for both spins so that $\mathrm{1/2}{M}_{z}^{\ast }$ (and hence *T*_*c*_) shall decrease with increasing pairing strength in the BEC limit.

Upon our solutions, we shall also enforce a positive definite compressibility^18^, which has been shown to be equivalent to the following condition^19^:

7 $${\frac{{\partial }^{2}{{\rm{\Omega }}}_{S}}{\partial {{\rm{\Delta }}}^{2}}|}_{{\mu }_{\uparrow },{\mu }_{\downarrow }}\mathrm{=2}\sum _{{\bf{k}}}\frac{{{\rm{\Delta }}}^{2}}{{E}_{{\bf{k}}}^{2}}[\frac{1-2\bar{f}({E}_{{\bf{k}}})}{2{E}_{{\bf{k}}}}+\bar{f}\text{'}({E}_{{\bf{k}}})] > \mathrm{0,}$$

where $\bar{f}{\rm{\text{'}}}(x)={\rm{d}}\bar{f}(x)/{\rm{d}}{\rm{x}}$, and ${{\rm{\Omega }}}_{S}$ is the thermodynamic potential, whose formal expression can be found in ref.^20^. Phase separation may occur when this stability condition is not satisfied.

## Numerical Results and Discussions

**Figure 1:**
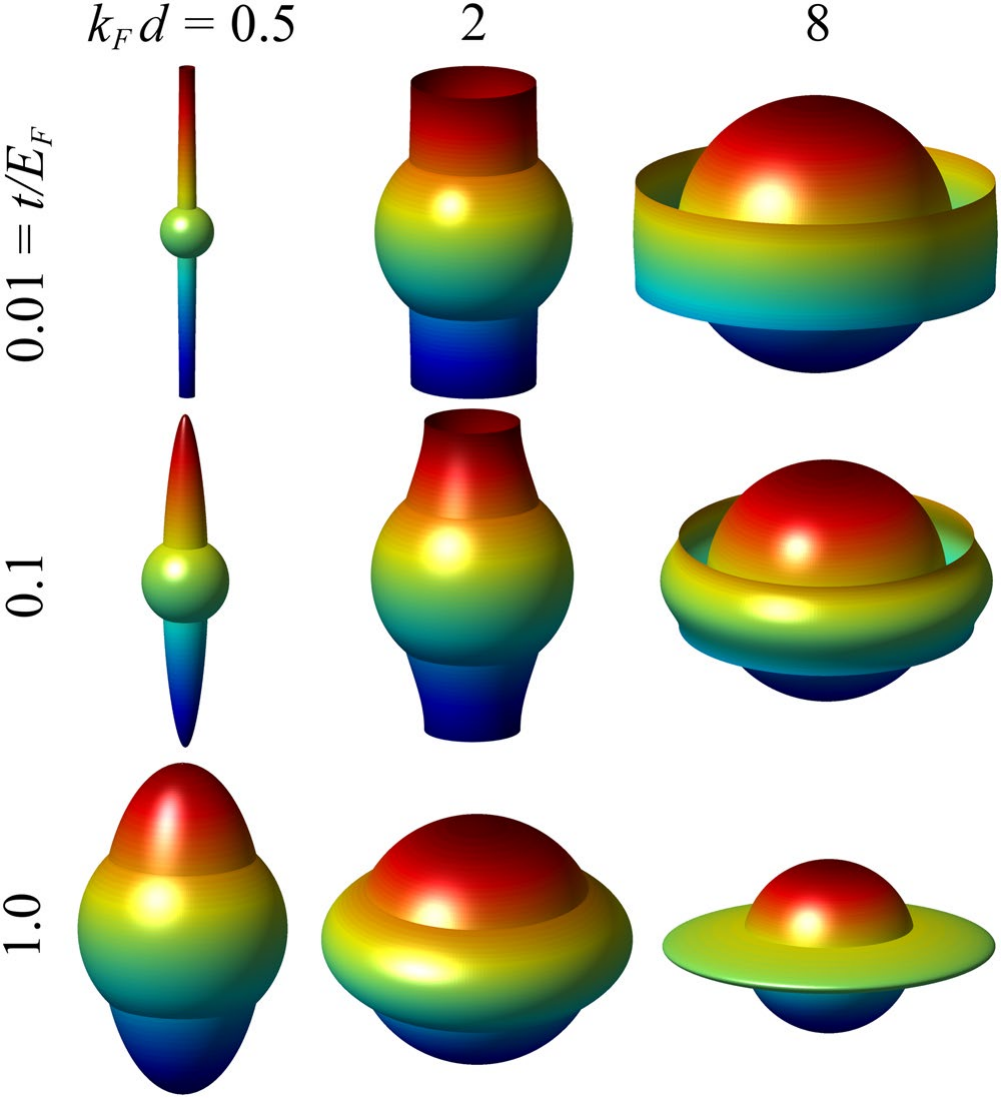
Evolution of the Fermi surface of the lattice component as a function of *t* and *d*, as compared with that of the 3D component (represented by the sphere). The Fermi surface is more like quasi-1D for small *d* and quasi-2D for large *d*.

Before we present our solutions on the phase diagrams, let’s first study the Fermi surface mismatch in the noninteracting limit. In Fig. 1, we show how the Fermi surface of the lattice component evolves as a function of *t* and *d*, as compared with the 3D component, which is represented by the sphere. The closest match occurs near $t/{E}_{F}=1$ and *k*_*F*_ *d* = 1 (not shown). For fixed *t*, the Fermi surface of the lattice component evolves from an elongated cigar shape (quasi 1D) to a pan cake or disc (quasi 2D), as *d* increases. On the other hand, for fixed *d*, the Fermi surface may change from a pan cake (quasi 2D) to a cigar or a long cylinder (quasi 1D), as *t* decreases. This can be readily understood. When *t* is small, it is more energetically favorable to populate on the ${k}_{z}$ quantum levels than the in-plane ${k}_{\parallel }$ levels. However, if *d* is large, the first band $|{k}_{z}| < \pi /d$ becomes quickly filled so that fermions have to accumulate in high ${k}_{\parallel }$ levels, leading to a disc-like Fermi surface for small *t* and large *d*. It is this case which is mostly relevant to real experimental configurations, which may be expected to satisfy $t{d}^{2} < 1/2m$.

Figure 1 reveals that large Fermi surface mismatch may occur for large and small $(d,t)$. We shall now see this mismatch effect at the mean-field level first.

**Figure 2:**
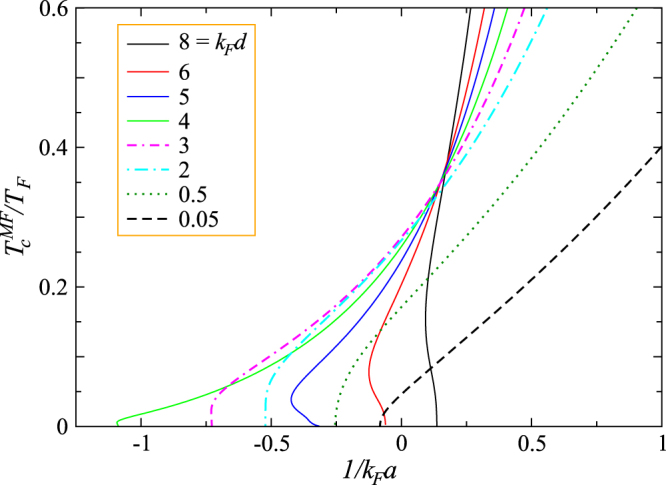
Mean-field solution of ${T}_{c}^{MF}$ as a function of $1/{k}_{F}a$ for different *d* with $t/{E}_{F}=0.05$. Intermediate temperature superfluidity occurs for *k*_*F*_ *d* = 5, 6 and 8.

Mean-field solutions can be obtained by solving Eqs (2)–(4), assuming that the gap is the order parameter. Shown in Fig. 2 are a series mean-field *T*_*c*_ curves as a function of $1/{k}_{F}a$ with different *d* and fixed $t/{E}_{F}=0.05$. For this small value of $t$, the best Fermi surface match occurs near *k*_*F*_ *d* = 4, in which case, the ${T}_{c}^{MF}$ curves to the left most into the BCS regime. As *d* increases (solid lines) or decreases (dashed lines), the curves, esp. their low *T* thresholds, move towards stronger coupling. In other words, these large or small *d* values have stronger pair breaking effects at low *T* so that stronger pairing strength is needed to achieve pairing. For *k*_*F*_ *d* > 4, there is clear evidence for intermediate temperature superfluidity, as found in conventional population imbalanced Fermi gases in a simple 3D continuum^15^.

**Figure 3:**
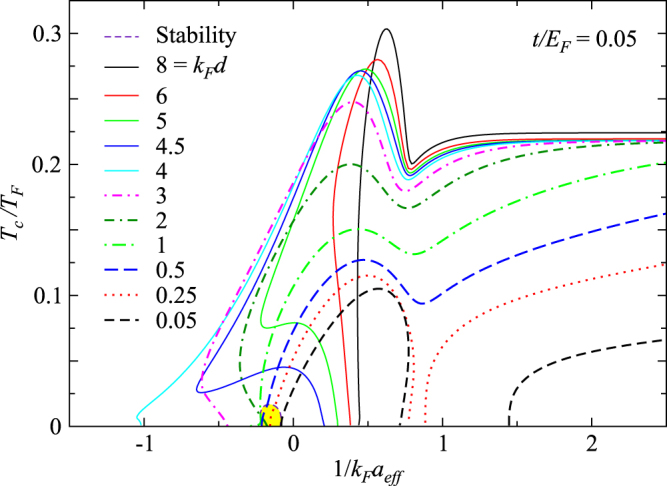
Behavior of *T*_*c*_ as functions of $1/{k}_{F}{a}_{eff}$ at fixed *t*/*E*_*F*_ = 0.05, but for different value of *k*_*F*_ *d* from 8 to 0.05. The *T*_*c*_ solution in shaded regions is unstable against phase separation.

We now proceed and present our main result with pairing fluctuation effects included. While the $(t/{E}_{F},{k}_{F}d)=\mathrm{(1},\mathrm{1)}$ possess the highest Fermi surface match, such a large *t* value is hard to realized experimentally. As a reference, we present this case in Supplementary Fig. S1. Here we present in Fig. 3 a more realistic case of $t/{E}_{F}=0.05$, and plot *T*_*c*_ as a function of $\mathrm{1/}{k}_{F}{a}_{eff}$ for a series of *d* from large to small. For this case, the best Fermi surface match occurs near *k*_*F*_ *d* = 4 (See Fig. 1), for which the *T*_*c*_ curve extends the deepest into the BCS regime, similar to the mean-field case. As *d* becomes smaller (dashed lines), the threshold for the *T*_*c*_ curve moves to the right, similar to the mean-field result, and the *T*_*c*_ values are suppressed at the same time. For *k*_*F*_ *d* = 0.25 and smaller (0.05), *T*_*c*_ is pinched and split into two parts at intermediate coupling strength, in the regime around *μ* = 0, exhibiting a re-entrant superfluidity.

**Figure 4:**
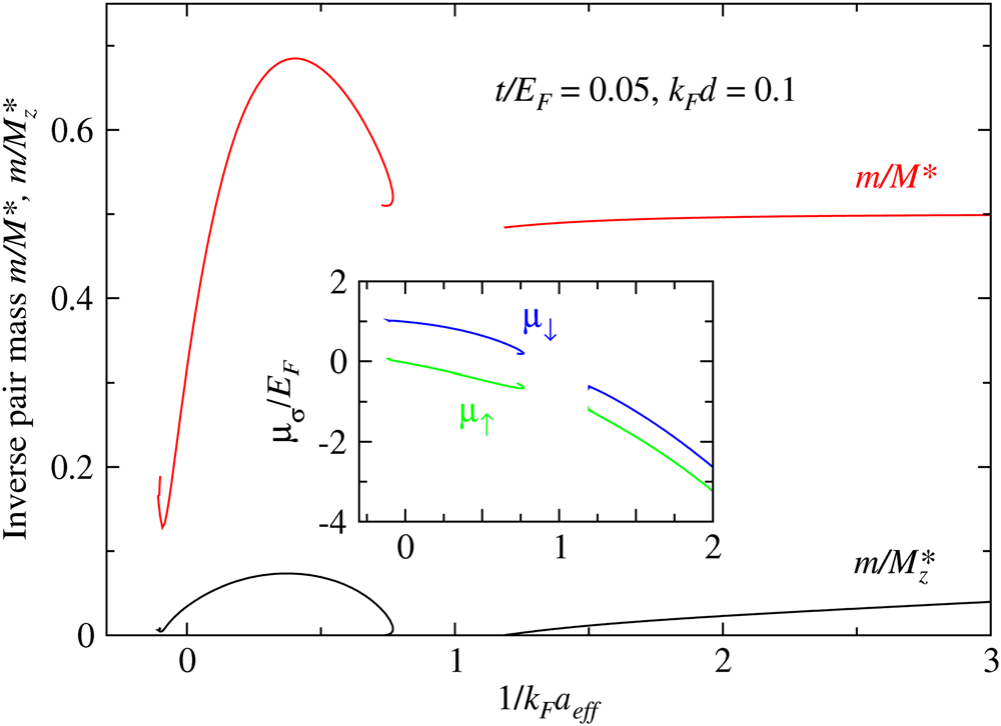
Behavior of the in-plane (red) and out-of-plane (black) components of the inverse pair masses (main panel) and chemical potentials (inset, *μ*_↑_ and *μ*_↓_, as labeled) as a function of $\mathrm{1/}{k}_{F}{a}_{eff}$, for *t*/*E*_*F*_ = 0.05 and *k*_*F*_ *d* = 0.1. The sign changes in *m/Mz* ^*^ lead to pair density wave ground state in between, exhibiting reentrant superfluidty. Here *M* ^*^ ≡ *M*_ǁ_^*^.

Such a re-entrant *T*_*c*_ behavior was previously seen in the crossover regime in a dipolar Fermi gas^21^. This is a regime which interpolates the BCS and the BEC regimes, where real space pairs start to emerge as well defined composite particles while the inter-pair repulsive interaction is very strong. For the present case, as can be seen from Fig. 1, the highly elongated quasi-1D Fermi surface of the lattice component for small *d* causes a large Fermi surface mismatch. This mismatch then strongly suppresses the mobility of the pairs in the $\hat{z}$ direction, leading to possible Wigner crystallization of the pairs, and hence a pair density wave (PDW) ground state without superfluidity. The Wigner crystallization is signaled by a sign change of the effective pair mass at zero momentum, as shown in Fig. 4. In the PDW state, the pair dispersion would reach its minimum at a finite momentum. Such a potential energy driven PDW state should not be confused with an FFLO states. The plot of ${\mu }_{\sigma }$ in the inset of Fig. 4 reveals that the chemical potential for the lattice component is very small in size in the BCS regime for this small value of *k*_*F*_ *d*.

**Figure 5:**
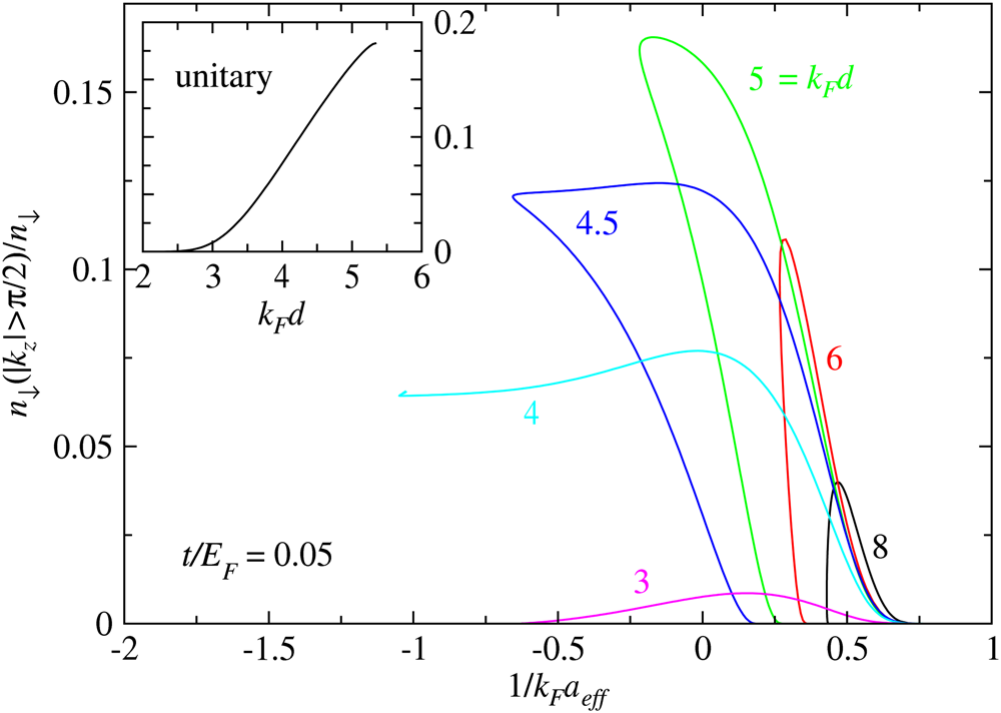
Fraction of the 3D component outside the first Brillouin zone along the *T*_*c*_ curves with $t/{E}_{F}=0.1$ for different values of *k*_*F*_ *d*. The inset plots the fraction at *T*_*c*_ as a function of *k*_*F*_ *d* at unitarity. The fraction increases with *k*_*F*_ *d* but vanishes for all cases in the BEC regime.

On the other hand, as *k*_*F*_ *d* increases from 4 (solid lines in Fig. 3), the lattice Fermi surface becomes a disc, and the lattice component becomes more 2D like. While this also leads to a large Fermi surface mismatch, its damage can be substantially alleviated when the pairing interaction becomes strong, since pairing effectively prevents the 3D component from occupying large $|{k}_{z}|$ states, making it a quasi-2D system as well. To see this, we plot the fraction of the 3D component with $|{k}_{z}| > \pi /d$. This effect is manifested in Fig. 5, where we plot this fraction as a function of $\mathrm{1/}{k}_{F}{a}_{eff}$ for different *d*’s, calculated along the *T*_*c*_ curves. It is obvious that the fraction increases with *d* for given $\mathrm{1/}{k}_{F}{a}_{eff}$. (Shown in the inset is a continuous curve as a function of *d* for the unitary case). A large fraction results from a large Fermi surface mismatch. As $\mathrm{1/}{k}_{F}{a}_{eff}$ progresses into the BEC regime, this fraction quickly decreases to zero. Therefore, in the BEC regime, all the large *d* curves quickly converge and approach the BEC asymptote. However, in the BCS regime, the detrimental effect of the mismatch causes *T*_*c*_ to bend back towards stronger interaction in the low *T* regime. (For *k*_*F*_ *d* > 5.4, one loses superfluidity completely at $1/{k}_{F}a < 0$). For *k*_*F*_ *d* = 4, this fraction remains sizable as *T*_*c*_ vanishes in the BCS regime; this is the case for which the Fermi surface mismatch is nearly the least, so that superfluidity is allowed with such a small mismatch. Except for the *k*_*F*_ *d* = 4 case, for all other large *d* cases in Fig. 5, the fraction drops to zero as the *T*_*c*_ curves bend back towards BEC and decrease to 0. This suggests that pairing has to be strong enough so as to pull all down spin fermions back into the first Brillouin zone, in order to have a superfluid at zero *T*.

**Figure 6:**
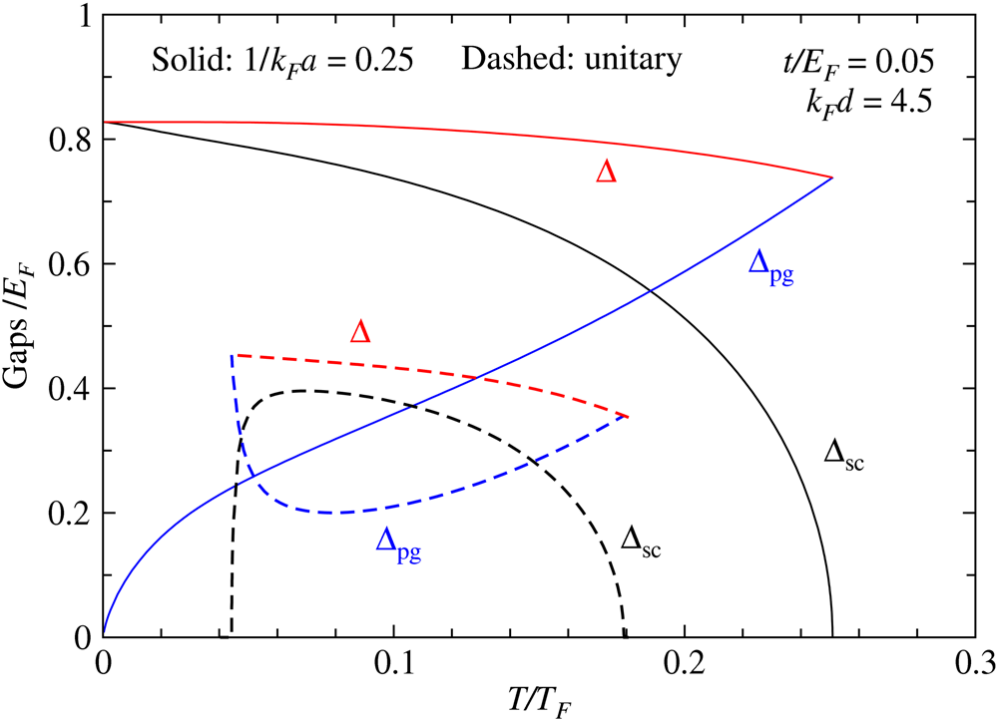
Typical behaviors of the order parameter ${{\rm{\Delta }}}_{sc}$ (black) and the gaps Δ (red) and ${{\rm{\Delta }}}_{pg}$ (blue curves), as a function of $T/{T}_{F}$ for $1/{k}_{F}a=0.25$ (solid) and unitary (dashed lines), representing cases without and with intermediate temperature superfluidity, respectively. Here *t*/*E*_*F*_ = 0.05 and *k*_*F*_ *d* = 405, as labeled.

The back bending of *T*_*c*_ at large *d* leads to a pronounced intermediate temperature superfluid behavior. We show in Fig. 6 representative behaviors of the gaps (Δ, Δ_*pg*_) and superfluid order parameter Δ_*sc*_ as a function of $T/{T}_{F}$, for the case with (dashed) intermediate temperature superfluidity, and compare with the case without (solid lines). For the former case, the order parameter vanishes at both lower and upper *T*_*c*_’s, sandwiched by pseudogap phases above and below.

We have also shown in Fig. 3 the (yellow shaded) area in which the *T*_*c*_ solution is unstable against phase separation. In comparison with the phase diagram of Fermi gases in a simple 3D continuum in the presence of population imbalance^15^, this unstable area is very small. We notice that this area exists only for small *d* cases. For large *d*, when *T*_*c*_ becomes nonzero, the Fermi surface mismatch is already alleviated by pairing.

**Figure 7:**
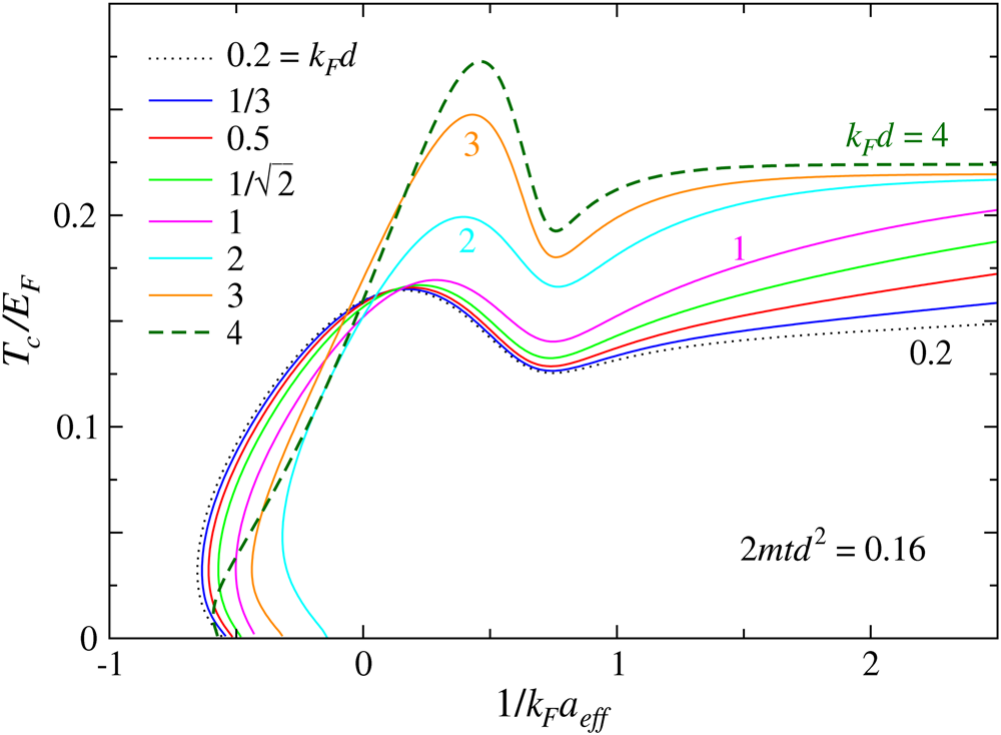
Behavior of *T*_*c*_ as a function of $1/{k}_{F}a$ for fixed $2mt{d}^{2}=0.16$. Except for large *d*, the *T*_*c*_ curves are close in the BCS and crossover regimes, while the discrepancies become more pronounced in the BEC regime. The *k*_*F*_ *d* = 4 case has a good Fermi surface match, exhibiting the least frustration on pairing.

Finally, we investigate the behavior of *T*_*c*_ with a fixed $m/{m}_{z,\uparrow }^{\ast }=2mt{d}^{2}$ but different $(t,d)$ combinations. This corresponds to fixed long wave length effective mass of the lattice component in the $\hat{z}$ direction. The curves would collapse to each other should the low *k*_*z*_ part of spin up fermions dominate the *T*_*c*_ behavior. Shown in Fig. 7 is a case with a small $m/{m}_{z,\uparrow }^{\ast }=0.16$, which is realistic for experiment. While the curves more or less converge in the fermionic regime, they separate on the BEC side of the Feshbach resonance. In the BCS regime, for small *d*, $\pi /d\gg {k}_{F}$, therefore, the lattice effect is not strong. In contrast, in the BEC regime, the BCS coherence factor ${v}_{{\bf{k}}}^{2}$ (i.e., momentum space pair occupation number) spreads throughout the entire *k*_*z*_ space, making the optical lattice effect fully probed. When *d* is large, say, $\pi /d < {k}_{F}$, lattice effect will be easily probed even in the BCS regime, leading to a more pronounced departure.

## Conclusions

In summary, we studied the behavior of the entire BCS-BEC crossover at finite temperature in mixed-dimensional Fermi gases using a pairing fluctuation theory. We found that tunable mixed dimensionality can create large Fermi surface mismatches. The *T*_*c*_ solutions bear similarity with simple population imbalance Fermi gases in a 3D continuum, but with some distinct features. While intermediate temperature superfluidity also exists, reentrant superfluid behavior with a pair density wave ground state in between emerges at small *d*. Unlike an pure optical lattice case, *T*_*c*_ approaches a constant asymptote in the deep BEC regime. With modern techniques, these predictions can be tested experimentally.

## Electronic supplementary material

Supplementary Information
