# Supplementary material for: Exotic superfluidity and pairing phenomena in atomic Fermi gases in mixed dimensions
Source: Sci Rep. 2017 Oct 11;7:12948. doi: 10.1038/s41598-017-13321-3 (PMC5636804; doi:10.1038/s41598-017-13321-3)
Supplement: Supplementary file 1 — Supplementary Information [file 41598_2017_13321_MOESM1_ESM.pdf]

Supplementary Information
Exotic superfluidity and pairing phenomena in atomic Fermi gases in mixed dimensions

Leifeng Zhang,[1,2] Yanming Che,[1,2] Jibiao Wang,[1,2,3] and Qijin Chen[1,2,4,*]

[1]*Department of Physics and Zhejiang Institute of Modern Physics, Zhejiang University, Hangzhou, Zhejiang 310027, China*
[2]*Synergetic Innovation Center of Quantum Information and Quantum Physics, Hefei, Anhui 230026, China*
[3]*TianQin Research Center & School of Physics and Astronomy, Sun Yat-Sen University (Zhuhai Campus), Zhuhai, Guangdong 519082, China*
[4]*James Franck Institute, University of Chicago, Chicago, Illinois 60637, USA*

(Dated: June 24, 2017)

Here we present extra plots which may help with the understanding of the main text.

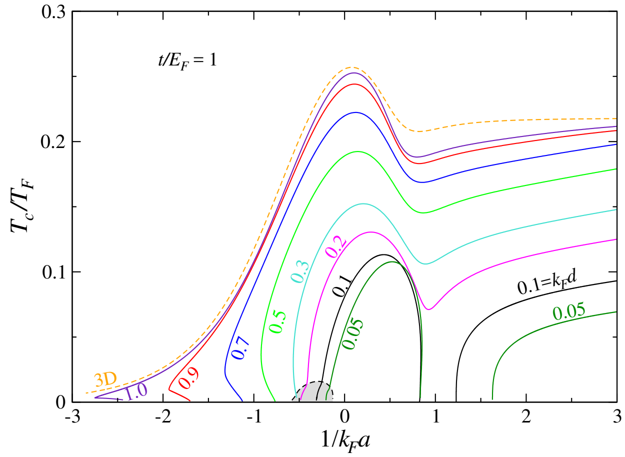

Figure S1. Behavior of T_c as functions of $1/k_F a$ at fixed $t/E_F = 1$, but for different value of $k_F d \leq 1$, as labeled. The T_c solution in shaded regions is unstable against phase separation.

SUPERFLUID TRANSITION T_c AS A FUNCTION OF $1/k_F a$ FOR $t/E_F = 1$

Shown in Fig. S1 is T_c as a function of $1/k_F a$ for $t/E_F = 1$, with a series of values of $k_F d$, as labeled. As a basis for comparison, we also included the T_c curve from a simple isotropic 3D Fermi gas, labeled "3D". For this large $t = E_F$, the best Fermi surface match occurs near $k_F d = 1$. Here we only show the curves with $k_F d < 1$, which do not intersect the 3D curve. The T_c curve splits for small d, giving way to pair density wave ground states. In the shaded area, the system is unstable at T_c. Intermediate temperature superfluid exists for $k_F d \geq 0.3$.

EFFECTS OF A BAND DISPERSION FOR PAIRS

To check the effect of a band dispersion for the pairs on T_c, we performed T_c calculations using both parabolic and band dispersions for the $\hat{z}$ direction of spin-up fermions. The result is shown in Fig. S2, for $t/E_F = 0.05$ and $k_F d = 4$. It is evident that the two curves overlap with each other for $1/k_F a < 0$, and only a minor quantitative difference arises in the BEC regime.

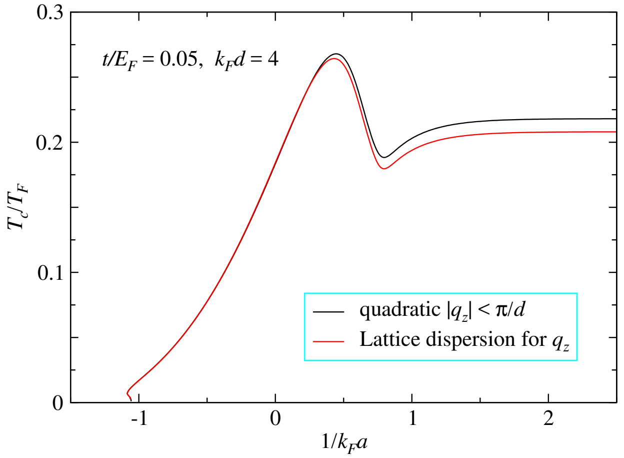

Figure S2. Comparison between two T_c solutions as a function of $1/k_F a$ using a parapolic dispersion (black) and a band dispersion (red) for the q_z contribution of the pair. Here $t/E_F = 0.05$ and $k_F d = 4$, as labeled.
